# Evaluation of large language model–generated information in diabetes health patient education: a scoping review

**DOI:** 10.3389/fpubh.2026.1850486

**Published:** 2026-06-23

**Authors:** Shiya Hu, Binfen Lin, Fenglan Lin

**Affiliations:** 1 Department of Education, The Affiliated People's Hospital of Fujian University of Traditional Chinese Medicine, Fuzhou, Fujian, China; 2 School of Nursing, Fujian University of Traditional Chinese Medicine, Fuzhou, Fujian, China; 3 Department of Respiratory and Critical Care Medicine, Lishui Central Hospital, Lishui, Zhejiang, China

**Keywords:** artificial intelligence, diabetes, education, large language model, scoping review

## Abstract

**Background:**

Diabetes is a leading cause of disability and death, posing a heavy healthcare burden globally. While standardized health education is crucial for glycemic control and mitigation of complications, traditional educational models face challenges due to insufficient scalability. The ongoing development of AI-based large language model (LLM) methods and technologies presents significant opportunities for health education in the field of diabetes.

**Objective:**

A scoping review of research on LLM-generated information for diabetes patient health education: Synthesizing current application status and performance outcomes.

**Methods:**

The Joanna Briggs Institute (JBI) evidence-based healthcare centre's scoping review guidance was utilized as the methodological framework, then five databases (PubMed, Embase, Web of Science, (American Psychological Association) APA PsycNet, and The Cochrane Library) were searched to retrieve studies from their inception to March 26, 2026. Two reviewers independently performed literature screening, full-text reading, and data extraction.

**Results:**

A total of 21 studies from nine countries were included. Application scenarios were categorized into five domains: general health education, dietary education, complication education, exercise education and technology education. Overall, the existing evidence indicates that LLMs perform well in terms of accuracy and completeness; however, significant limitations remain in readability, reliability, and usability. Moreover, ethical and safety concerns are prominent, including data security, fairness, patient safety, and liability.

**Conclusion:**

Despite existing technical and ethical challenges, LLMs still have potential as an auxiliary tool in diabetes health education. Future research needs to enhance technical design optimization, develop patient-centered designs, standardize evaluation metrics, and structured ethical oversight to further validate their practical application effects in diabetes health education for patients with diabetes.

## 1 Introduction

According to the latest data from the International Diabetes Federation, diabetes affects approximately 11.11% of the global adult population, with about 589 million adults suffering from the condition (1). Diabetes facilitates devastating complications, notably diabetic nephropathy and foot ulcers, which elevate morbidity and mortality while severely diminishing quality of life. Consequently, this creates a profound economic burden on both families and society. The American Diabetes Association (ADA) underscores that diabetes education and self-management support (DSMS) serve as the cornerstone of clinical care, significantly delaying the onset of complications and optimizing long-term health outcomes (2). However, the accessibility and equity of diabetes education face greater challenges due to issues such as uneven distribution of diabetes health resources and a shortage of professional medical personnel (3). Therefore, addressing the urgent need for scalable and high-quality health education resources is essential to bridge the current gaps in public health.

Artificial intelligence, as a new type of health technology, is gradually being applied to health education for patients with chronic diseases (4). In the landscape of artificial intelligence, large language models (LLMs) have become a research hotspot. LLMs are trained on massive amounts of text data to construct huge neural network structures, thereby processing complex semantic information and showing great potential in answering patient questions and providing health education (5). In the field of diabetes education, LLMs can provide health education to patients remotely and in real time (6). At the same time, LLMs can develop personalized health education materials for different patients, delivering knowledge to patients through open dialogue (7). However, evidence from existing studies reveal discrepancies in the performance of various LLMs concerning the reliability, accuracy, and readability of generated health education materials. Yigit Yalcin et al. (8) pointed out that Grok-generated health materials have the highest reliability, Wang et al. (9) indicated that ChatGPT-generated materials have even higher reliability.

As LLMs increasingly redefine the production of health education materials, a systematic analysis to delineate current challenges is imperative. Therefore, this study employs a scoping review approach to systematically map and summarize the application of LLMs in diabetes health education. By synthesizing the evaluative findings from existing literature regarding the quality of model-generated information, this study identifies current challenges and provides an evidence-based foundation for the future implementation of LLM-assisted health education.

## 2 Methods

The methodological framework for this review is informed by the JBI guidance for scoping reviews, and the reporting follows the PRISMA-ScR checklist (Supplementary Appendix A) (10, 11). All authors involved in the evidence synthesis received specialized training based on the Joanna Briggs Institute Reviewer's Manual 2020. In alignment with open science principles, the project was pre-registered on the Open Science Framework with the following identifier: 10.17605/OSF.IO/H4DZN.

### 2.1 Identify the research question

This review explores the role of LLMs in generating information to support diabetes health education, providing a comprehensive overview and critical evaluation. Specifically, it systematically synthesizes the applications of LLMs in providing diabetes health education and addresses the following issues:

(1) What specific LLMs have been implemented in the context of diabetes health education, and what are their underlying architectural characteristics?
(2) What are the primary clinical application scenarios for LLM-generated information in diabetes education, and how is their performance evaluated across key metrics?
(3) What are the prevailing technical and ethical challenges hindering the integration of LLMs into diabetes care, and what are the projected future trends for their clinical deployment?

### 2.2 Search strategy

Relevant English literature was searched in PubMed, Embase, Web of Science, APA PsycNet, and The Cochrane Library databases using keywords such as large language model, ChatGPT, transformer model, generative AI, and diabetes. The search period was from the database inception to March 26, 2026. Specific search strategies are detailed in Supplementary Appendix B.

### 2.3 Inclusion criteria

The inclusion criteria for the literature are:

(1) Target Audience: Health education materials and inquiries specifically designed for patients with diabetes mellitus and their caregivers.
(2) Concept: Generation of diabetes-related educational content by LLMs, including but not limited to ChatGPT, DeepSeek, Gemini, and Claude.
(3) Outcomes: Multidimensional performance metrics of the generated information, such as clinical accuracy, linguistic readability, reliability, and actionable guidance.

The exclusion criteria for literature are as follows:

(1) Studies that only describes the development of LLM technology;
(2) Studies that does not involve application scenarios of diabetes;
(3) Studies that is published repeatedly;
(4) Studies for which the full text cannot be obtained;
(5) Non-English studies.

### 2.4 Study selection and data extraction

Studies were imported into EndNotes software, and duplicates were removed. Two researchers (Shiya Hu and Binfen Lin) independently conducted initial and secondary screenings of the studies based on the inclusion and exclusion criteria. Disagreements were resolved through discussion or by consulting a third party (Fenglan Lin) for arbitration. This study employed the JBI data extraction and analysis framework. Information from the included studies was extracted using Excel: authors, year, country, study population, target end-users, applications, evaluation methods, AI tools, technical architecture, ethical considerations, main conclusion, and research results. This process was completed by two independent researchers and cross-validated by a third researcher to minimize bias.

## 3 Results

### 3.1 Literature review

**Figure 1:**
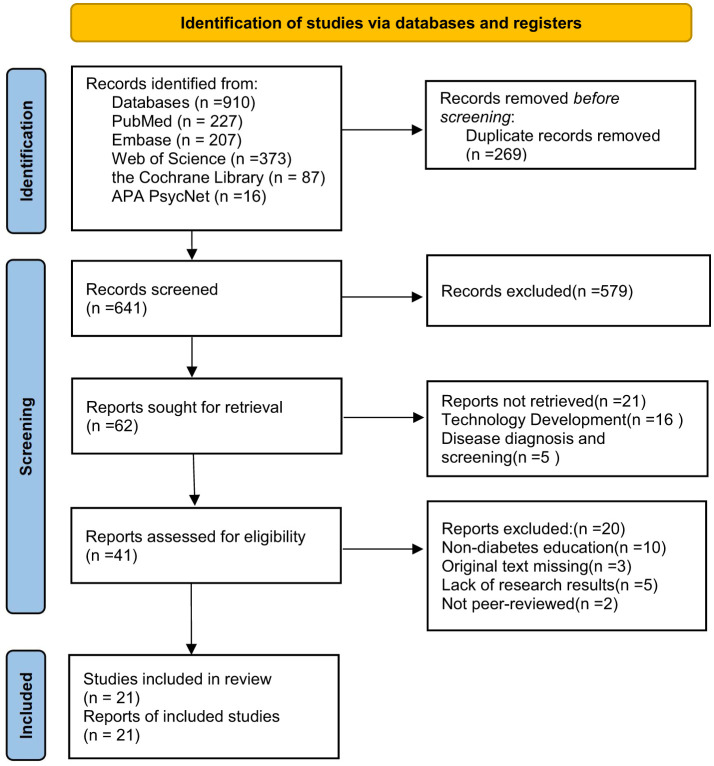
PRISMA flow chart.

The initial search yielded 910 studies, 641 of which remained after removing duplicates. After reviewing titles and abstracts, 579 studies were selected, leaving 62 studies for full-text review. Following the full-text assessment, a further 41 studies were removed, resulting in a final inclusion of 21 studies. The detailed screening process is shown in Figure 1.

### 3.2 Study characteristics

All 21 studies included in this review were published between 2023 and 2026. These studies span nine countries, with the highest representation from Turkey (*n* = 7) (8, 12–17) and China (*n* = 5) (9, 18–21), followed by the United States (*n* = 2) (22, 23), Denmark (*n* = 2) (6, 24), and one study each from South Korea (25), India (26), Norway (27), Ireland (28), France (29). Regarding the distribution of AI platforms across the included studies, ChatGPT was the most extensively evaluated model, featured in all 21 studies (100%). Other prominent LLMs investigated included Gemini (28.57%, *n* = 6) (8, 13, 14, 16, 19, 29), DeepSeek (23.81%, *n* = 5) (8, 9, 15, 16, 20), Claude (14.29%, *n* = 3) (9, 19, 29), Grok (9.52%, *n* = 2) (8, 15). Additional models mentioned in single studies (4.76% each) included ERNIE Bot (18), Google Bard (19), Doubao (20), DanskGPT (6). A detailed breakdown of the specific LLM versions utilized in each study is presented in Table 1.

**Table 1:** The characteristics of the included studies.

References	Country	Population	Target end-users	Applications	Evaluation methods	AI tools	Technical architecture	Ethical considerations	Main conclusion
Subramanian et al. (26)	Indian	Diabetes	Patients	Q&A	Expert	ChatGPT-4	/	Potential bias	ChatGPT has high accuracy in treating diabetic retinopathy.
Chung et al. (25)	Korea	Type 2 diabetes	Patients	Q&A	Expert	ChatGPT-4	/	/	ChatGPT can serve as a supplementary resource for exercise in diabetes. However, ChatGPT may not provide complete answers to some questions about exercise for diabetes.
Tekin et al. (12)	Turkey	Diabetes	Patients, clinicians	Q&A	Expert	ChatGPT-4o		Information obsolescence, fairness, and source transparency	ChatGPT-4o offers medium to high reliability and quality, but has limited readability.
Cheng et al. (18)	China	Diabetic kidney disease	Patients, clinicians	Health education content creation	Expert, patient	ERNIE Bot 4.0, ChatGPT-4o, ChatGLM4, ChatGPT-o1		Information accuracy, security, and attribution of responsibility	Results indicated that ERNIE Bot 4.0 demonstrated non-inferiority to human experts, delivering health education content that matched clinician-authored materials in understandability, completeness, and safety profiles.
Skjervold et al. (27)	Norway	Diabetes	Patients	Q&A	Patient	ChatGPT-4o	/	Error message generation, data privacy and security, and liability attribution	ChatGPT-4o scored significantly higher than human healthcare professionals in all three dimensions: knowledge accuracy, usefulness, and empathy.
Kelly et al. (28)	Ireland	Diabetes	Patients	Q&A	Expert	ChatGPT-4o mini	RAG	/	LLM chatbots based on RAG can provide context-aware, empathetic, and clinically reliable answers to T2DM queries.
Wang et al. (19)	China	Diabetes	Patients, clinicians	Q&A	Expert, patient	ChatGPT-4, RAG-ChatGPT, Anthropic Claude 2, RAG-Anthropic Claude 2, Google Bard, RAG-Google Bard	RAG	/	The integration of RAG substantially enhanced the performance of LLMs in addressing diabetes-related inquiries, yielding significant improvements in accuracy, comprehensiveness, and understandability.
Zha et al. (20)	China	Diabetes	Clinicians	Health education content creation	Expert	ChatGPT-4o, Doubao 1.5, DeepSeek R1	/	/	ChatGPT-4o, Doubao 1.5, and DeepSeek R1 can all generate accurate and easy-to-understand materials, but their operability and safety remain questionable.
Wang et al. (9)	China	Gestational diabetes mellitus	Patients, clinicians	Q&A	Health education content creation	ChatGPT-5, ChatGPT-4o, DeepSeek-V3.2, DeepSeek-R1, Gemini 2.5 Pro, Claude Sonnet 4.5	/	/	LLMs can generate GDM-related information that is generally accurate and moderately reliable, with next-generation models showing significant improvements in diagnostic validity. However, limited transparency and highly systematic reading volumes suggest that these tools are not yet suitable as independent resources for GDM patient education and should be used as supplementary materials for clinical consultation and professional planning.
Hernandez et al. (22)	United States	Diabetes	Patients	Q&A	Expert	ChatGPT	/	/	ChatGPT can provide high-quality, reliable medical information.
Yigit Yalcin et al. (8)	Turkey	Gestational diabetes mellitus	Patients	Q&A	Expert	ChatGPT-4o, Gemini 2.5 Pro, Grok 3.0, DeepSeek R-1	/	Data privacy, fairness, and patient safety risks	LLMs can provide moderately reliable content with varying readability, but their output may not be sufficient for unsupervised patient use.
Ongen et al. (13)	Turkey	Type 1 diabetes in children	Caregivers	Q&A	Expert	ChatGPT-3.5, ChatGPT-4, ChatGPT-4o, Gemini, Gemini advanced	/	/	All the LLMs evaluated performed similarly in answering common questions about type 1 diabetes.
Sun et al. (21)	China	Type 2 diabetes	Patients	Q&A	Expert	ChatGPT, GPT 4.0	Multimodal Fusion	/	LLMs can provide food recommendations that largely conform to best practices and have high accuracy.
Lindstrøm et al. (6)	Denmark	Gestational diabetes mellitus	Patients	Q&A	Expert	ChatGPT-v4, DanskGPT	/	/	LLMs hold considerable promise as supportive clinical resources, offering a scalable solution for patient education and inquiry management within the domain of gestational diabetes.
Aypar Akbag (14)	Turkey	Gestational diabetes mellitus	Patients	Health education content creation	Expert	ChatGPT, Gemini	/	/	GDM educational content produced by ChatGPT and Gemini exhibited superior readability, ensuring accessibility for patients with varying levels of health literacy.
Bayram et al. (15)	Turkey	Diabetes	Patients	Health education content creation	Expert	ChatGPT-4.1, Grok-3, DeepSeek-in	/	Information accuracy, privacy risks, hallucinations, and attribution of responsibility	None of the models fully aligned with the Nutrition Care Process framework, showing significant deficiencies particularly in the “Diagnosis” and “Monitoring/Evaluation” domains. Furthermore, the outputs were hindered by frequent hallucinations and a profound lack of clinical contextualization.
Johansen et al. (24)	Denmark	Adolescents with type 1 diabetes	Patients	Q&A	Expert	ChatGPT-4o	Multimodal fusion	/	ChatGPT demonstrated high accuracy in estimating the carbohydrate content of single-item foods, such as fruits and vegetables; however, its performance declined significantly when evaluating complex mixed meals.
Senoymak et al. (17)	Turkey	Diabetes	Patients	Q&A	Expert	ChatGPT-3.5	/	/	ChatGPT exhibited proficient performance in delivering accurate health information for diabetes, showcasing its potential as a reliable digital health resource.
Rohrich et al. (23)	United States	Diabetic foot ulcer	Patients	Q&A	Expert	ChatGPT-3.5	/	/	ChatGPT is effective at providing general information, but it can provide incorrect information. The problem of omitting key details
Goncalves et al. (29)	France	Type 1 diabetes	Patients	Q&A	Expert	ChatGPT-4o, Gemini 2.5 flash, Claude Sonnet 4	Multimodal Fusion	/	LLMs can provide carbohydrate estimates, but their accuracy varies.
Özkaya s et al. (16)	Turkey	Type 1 diabetes	Patients, clinicians	Q&A	Expert	ChatGPT-4.0, DeepSeek, Gemini	Multimodal Fusion	/	The accuracy of LLMs in carbohydrate counting was comparable to that of professional registered dietitians; however, significant heterogeneity was observed across different LLM platforms.

Q&A, clinical question answering; RAG, retrieval-augmented generation.

The end-user types covered in this review include patients (6, 8, 9, 12, 14–19, 21–29), caregivers (13), and clinicians (9, 12, 16, 18–21). The application forms of the included studies are primarily categorized into two areas: clinical question-answering (Q&A) (6, 8, 9, 12, 13, 16, 17, 19, 21–29) and the generation of health education materials (14, 15, 18, 20).

From a technical perspective, two studies (19, 28) leveraged Retrieval-Augmented Generation (RAG) to synthesize information, a strategy specifically employed to mitigate the inherent risk of “hallucinations” and enhance the clinical precision of health education content. Four studies (5, 16, 21, 24) adopted a multimodal fusion approach, facilitating nutritional education through the weighted integration and associative analysis of image pixel distribution and textual semantics. The remaining literature focused on the direct, zero-shot application of standalone LLMs without additional architectural enhancements.

The application of LLM-generated content in diabetes health education is primarily concentrated in three domains: health knowledge education (13, 17, 19, 20, 22, 27, 28), dietary education (5, 15, 16, 21, 24), complication education (6, 8, 9, 14, 18, 23, 26). Within the realm of complications, the studies specifically addresses gestational diabetes mellitus gestational diabetes (*n* = 4), diabetic retinopathy (*n* = 1), diabetic nephropathy (*n* = 1), and diabetic foot ulcers (*n* = 1). Conversely, sparse research has focused on exercise-related interventions (25) and technology-based education (12). Specific characteristics of the included literature are shown in Table 1.

The ethical dimensions of LLMs were explored across six studies (8, 12, 15, 18, 26, 27), focusing specifically on safety (*n* = 6), data privacy (*n* = 3), and accountability (*n* = 3). Notwithstanding these contributions, the existing studies are largely characterized by theoretical abstraction, with a notable dearth of concrete implementation frameworks. A comprehensive synthesis of the identified ethical challenges is provided in Supplementary Appendix C.

### 3.3 Study outcomes

Most included studies adopted a multidimensional approach to evaluate LLM performance. To ensure comparability across outcome measures, we established standardized definitions for the primary endpoints extracted from the included studies (Table 2).

**Table 2:** Outcome assessment of included studies.

Metrics	Assessment tools	Studies	Proportion of studies (%)
Accuracy	Likert scales	(6, 17–19, 23, 27)	61.95%
	Binary classification	(9, 15, 21, 24, 28)	
	Mean absolute error	(16, 29)	
	Artificial intelligence (AI) natural language evaluation tool	(20)	
Completeness	Likert scales	(17–19, 23, 26)	23.81%
Readability	The Gunning Fog Index (GFI)	(8, 9, 12, 14, 23)	28.57%
	Flesch reading ease (FRES)	(8, 9, 12, 23)	
	Flesch–Kincaid Grade Level (FKGL)	(8, 9, 12)	
	Coleman–Liau index (CL)	(8, 9, 23)	
	Simple Measure of Gobbledygook (SMOG index)	(8, 9, 23)	
	Likert scales	(8, 19)	
	The Automated Readability Index (ARI)	(9, 23)	
	The Atesman Readability Formula (ARF)	(14)	
	Linsear write formula, new Dale-Chall score	(23)	
Quality	The Global quality scale (GQS)	(8, 9, 12, 13)	19.05%
	DISCERN scale (mDISCERN)	(8, 9, 12)	
	The ensuring quality information for patients (EQIP)	(9)	
Appropriateness	Binary classification	(22, 28)	14.29%
	Likert scales	(26)	
Comprehensibility and actionability	Materials assessment tool for print materials (PEMAT-P)	(14, 20)	9.52%
Safety	Likert scales	(18, 23, 25)	19.05%
	Artificial intelligence (AI) natural language evaluation tool	(20)	
Utility	Likert scales	(12, 25)	14.29%
	Artificial intelligence (AI) natural language evaluation tool	(20)	
Validity	Likert scales	(25, 27)	9.52%
Empathy	Likert scales	(27)	4.76%
	Overall patient assessment Binary classification	(18)	4.76%
Personalization	Artificial intelligence (AI) natural language evaluation tool	(20)	4.76%

#### 3.3.1 Accuracy

Accuracy was defined as the factual correctness of the information generated by the LLMs. Across the included studies, various metrics were employed for assessment: six studies (6, 17–19, 23, 27) utilized Likert scales, five studies (15, 19, 21, 24, 28) adopted binary classification, and two studies (5, 16) applied Mean Absolute Error. For details, see Table 2. One study demonstrated that both DanskGPT and ChatGPT achieved accuracy levels comparable to clinicians, with ChatGPT significantly outperforming DanskGPT (*P* < 0.05) (6). Notably, ERNIE Bot 4.0 (Baidu Inc., Beijing, China) was reported to exceed clinicians in generating accurate information (18). Regarding nutritional precision, ChatGPT, DeepSeek, and Gemini showed no significant deviations from actual carbohydrate content (*P* > 0.05), with ChatGPT exhibiting the highest concordance with professional registered dietitians (16). In binary evaluations, the accuracy of ChatGPT was reported at 60.5%, whereas GPT-4.0 reached 74.5% (21). Furthermore, an assessment using an AI-based natural language evaluation tool indicated no significant performance gap between DeepSeek, ChatGPT-4o, and Doubao (*P* > 0.05) (20). However, inter-model heterogeneity in carbohydrate counting accuracy was highlighted in two studies (15, 29). Finally, LLMs integrated with RAG demonstrated superior accuracy compared to their baseline counterparts (19). Comprehensive details of these findings are summarized in Supplementary Appendix D.

#### 3.3.2 Readability

Readability reflects the ease with which readers can read and understand information generated by LLMs. Six studies (8, 9, 12, 14, 19, 23) assessed readability. Of these, five studies (8, 9, 12, 14, 23) used the Gunning Fog Index (GFI), four studies (8, 9, 12, 23) used the Flesch Reading Ease (FRES), four studies (8, 9, 12, 23) used the Flesch-Kincaid Grade Level (FKGL), three studies (8, 9, 23) used the Coleman-Liau Index (CL), three studies (8, 9, 23) used the Simple Measure of Gobbledygook (SMOG index), one study (19) used Likert scales, two studies (9, 23) used the Automated Readability Index (ARI), one study (14) used the Atesman Readability Formula (ARF), and one study (23) used the Linsear Write Formula and New Dale-Chall Score. Detailed assessment methodologies are summarized in Table 2. FRES assesses the ease of reading information generated by large language models; a higher score indicates greater ease of reading. FKGL, SMOG, GFI, ARI, and CL are used to assess the level of education required to read information generated by large language models. The American Medical Association (AMA) and the National Institutes of Health (NIH) recommend that patients align educational materials with a sixth-grade reading level. Therefore, an FRES ≥ 80.0 and FKGL, ARI, CL, GFI, and SMOG scores below 6 indicate good readability.

All six studies showed that the readability of information generated by large language models was poor and did not meet the recommendations of AMA and NIH. Comprehensive details of these findings are summarized in Supplementary Appendix D.

#### 3.3.3 Completeness

Completeness generally denotes the comprehensiveness of the generated information in addressing user inquiries or thematic areas. Five studies (17–19, 23, 26) used Likert scales to assess the completeness of information generated by LLMs. Detailed assessment methodologies are summarized in Table 2. One study found that ChatGPT-4 generated information that reached 87.6% of expert consensus (26). Another study indicated that Claude 2 generated the most complete information, followed by ChatGPT-4, while Google Bard had the worst completeness, and that augmentation systems improved the completeness of information generated by models across all models (19). Two studies indicated that ChatGPT-3.5 (OpenAI, San Francisco, CA, USA) generated information with good completeness (17, 23). However, the study pointed out that GPT-4o and ChatGLM4 generated health materials that differed significantly from those derived from physicians (18). Comprehensive details of these findings are summarized in Supplementary Appendix D.

#### 3.3.4 Quality

This dimension is designed to evaluate the comprehensive quality of the generated information, encompassing key metrics such as overall utility and reliability. Information quality was assessed across four studies (8, 9, 12, 13) using the Global Quality Scale (GQS). Specifically, three of these studies (8, 9, 12) utilized the DISCERN instrument to assess this metric, while one study (9) further integrated the Ensuring Quality Information for Patients (EQIP). Detailed assessment methodologies are summarized in Table 2. ChatGPT-4o was characterized as providing high-quality responses in one investigation (12). Performance variations were also observed across different model iterations; specifically, ChatGPT-4o outperformed both ChatGPT-4 and ChatGPT-3.5, while Gemini Advanced demonstrated superior quality compared to its standard Gemini counterpart (13). Currently, the quality of responses varies significantly across different LLM platforms. While one study identified ChatGPT as the top performer, with Gemini and Claude Sonnet showing poorer performance (9), divergent results were reported in another study where Gemini achieved the highest quality scores (8). Comprehensive details of these findings are summarized in Supplementary Appendix D.

#### 3.3.5 Comprehensibility and actionability

Understandability measures whether users can comprehend the information generated by LLMs, while actionability assesses whether the provided advice can be practically implemented. Two studies (14, 20) employed the Patient Education Materials Assessment Tool for Print Materials (PEMAT-P) for these evaluations. Detailed assessment methodologies are summarized in Table 2. One study reported that the average understandability score for ChatGPT and Gemini was 91.36% (86.66%−93.75%), with an average actionability score of 89.67% (80%−100%) (14) In a comparative evaluation of three LLMs, DeepSeek R1 achieved the highest scores for understandability, whereas ChatGPT-4o demonstrated the superior performance in terms of actionability (20). Comprehensive details of these findings are summarized in Supplementary Appendix D.

#### 3.3.6 Other evaluative metrics

Beyond core quality metrics, the six studies expanded their scope to encompass usability utility (*n* = 4) (12, 20, 25, 27), safety (*n* = 4) (18, 20, 23, 25), appropriateness (*n* = 3) (22, 26, 28), empathy (*n* = 1) (27) overall patient assessment (*n* = 1) (18) and personalization (*n* = 1) (20). Detailed assessment methodologies are summarized in Table 2. Regarding response alignment, one study reported a 96.8%concordance between ChatGPT-4o and expert clinicians (26), conversely, another study identified that 28.6%of ChatGPT-generated responses suffered from informational incompleteness (25). The evaluation of utility yielded mixed results: while one investigation found ChatGPT-4o's utility to be superior to that of healthcare professionals (27), another categorized its outputs as having only a moderate level of utility (12). In a comparative safety analysis of three LLMs, DeepSeek R1 achieved the highest safety rating, whereas Doubao 1.5 recorded the lowest safety scores (20). Comprehensive details of these findings are summarized in Supplementary Appendix D.

## 4 Discussion

As the first review to synthesize evidence on LLM-generated information for diabetes education, this study reveals a multifaceted performance profile. Synthesizing evidence from extant studies, LLM-generated health materials demonstrate commendable accuracy and comprehensiveness; however, they remain deficient in readability, actionability, and independent clinical utility. Although LLMs exhibit distinct advantages in generating diabetes-related educational content, they are currently not positioned to function as autonomous tools in diabetes health education.

### 4.1 Current status of research on information generation from LLMs in diabetes health education

Our analysis included 21 recent publications from the past 4 years, a trend that highlights the rapid emergence and scholarly interest in LLM-mediated health information. The inherent potential of LLMs in diabetes education stems from their robust frameworks for semantic comprehension, inferential reasoning, and high-fidelity content synthesis (30).

In terms of content distribution, general health education emerged as the most prevalent category. This trend likely reflects the extensive demand for foundational disease knowledge among the vast patient population, given the chronic and high-prevalence nature of diabetes. Dietary education represented the second most common type of information, which is consistent with the central role of nutritional management in diabetes self-care (31). Furthermore, the inclusion of complications—ranging from gestational diabetes to retinopathy, nephropathy, and foot ulcers—demonstrates the potential breadth of LLMs in multidimensional diabetes management. Nevertheless, research focusing on exercise (25) and technology-based education (12) remains sparse, indicating that these specialized areas are still in their nascent stages of exploration.

Notably, diabetes health education, particularly in the realms of diet and exercise, imposes more stringent requirements on LLMs. In dietary education, tasks frequently involve image input and recognition; however, model performance in vision-based tasks significantly lags behind text-processing capabilities, leading to compromised accuracy (24, 29). This disparity is primarily attributable to a deficit in sufficient visual feature descriptions within pre-training datasets, which hinders the model's ability to establish precise semantic correlations (32). Furthermore, exercise education necessitates dynamic adjustments based on real-time blood glucose levels, activity type, intensity, and duration (33). Such complexities demand advanced capabilities in personalized reasoning and real-time data integration. Moreover, the accelerating pace of technological innovation in diabetes care necessitates highly responsive knowledge update mechanisms, a domain where current LLMs remain vulnerable to information obsolescence (12).

In summary, the integration of LLMs into health education remains a double-edged sword. In some instances, the accuracy of LLM-generated outputs is comparable to educational materials developed by clinicians (6). However, superior technical performance fails to bridge the gap toward practical clinical application. This study highlights a critical deficit in the readability and actionability of LLM outputs. Specifically, the syntactic complexity of the generated materials often surpasses the recommended 6th-to-8th grade threshold, posing significant barriers for populations with lower health literacy. This misalignment aligns with a broader systemic bias in general LLMs during health dissemination (34, 35). Considering the vulnerability of diabetic patients to low digital literacy (36), suboptimal readability risks inducing a digital divide and deepening health inequities (37, 38). Furthermore, the lack of context-specificity undermines the models' actionability. In complex decision-making scenarios, LLMs tend to offer generalized, principle-based statements coupled with excessive redundancy (20), which limits practical application. Consequently, at this stage, LLMs are not yet suitable as standalone instruments but should instead serve as supervised auxiliary tools under the oversight of healthcare professionals.

At the technical level, due to the limited number of included studies, the impact of different architectural frameworks on generative quality remains insufficiently compared. Only one study (19) highlighted that RAG significantly enhances the accuracy, completeness, and understandability of LLM outputs. In the current body of literature, the majority of research continues to treat LLMs as “black-box” tools, with a notable lack of transparent reporting on critical parameters such as training datasets (9, 12, 19, 21). This technical opacity, to some extent, constrains the explainability and institutional trustworthiness of these models in clinical settings.

### 4.2 The practical impact and challenges of information generated by LLMs in diabetes health education

LLMs are increasingly being utilized to generate patient-facing health information, potentially reducing the administrative burden on healthcare providers while enhancing patient self-management capabilities (39). However, several critical barriers persist regarding the application of LLMs in health education.

First, the existing evaluation systems for LLM performance are characterized by a lack of standardization. Our analysis reveals that while 61.95% of studies assessed accuracy, essential clinical parameters including completeness, actionability, and safety remain severely under-reported (Table 2). Such gaps hinder a comprehensive validation of LLMs for clinical integration. In high-risk contexts such as diabetes management, the absence of rigorous assessment protocols may lead to unwarranted trust or erroneous interpretations of generated content (40). Moreover, significant heterogeneity exists in how performance metrics are defined and operationalized (41), compromising the comparability of findings and weakening the overall strength of evidence regarding LLM-based clinical interventions.

Comparatively, the transition of LLMs from controlled settings to real-world applications presents substantial hurdles. Current evidence is predominantly derived from pilot studies and simulated patient interactions, indicating a dearth of rigorous empirical verification in multifaceted, real-world contexts (25). Furthermore, existing literature has generally overlooked the influence of critical variables such as demographic characteristics and cultural contexts. However, significant heterogeneity exists among patients regarding disease progression, comorbidity burden, therapeutic regimens, digital health literacy, and socio-cultural backgrounds (42). Consequently, when confronted with real-world patient interactions, LLMs may still encounter issues such as poor information alignment, insufficient personalization, and unpredictable safety risks.

Lastly, six studies formally engaged with ethical considerations, with the majority offering only perfunctory descriptions rather than actionable frameworks. We have synthesized the ethical landscape into a four-fold typology: privacy, clinical safety, equity, and algorithmic accountability. As diabetes care necessitates persistent data engagement, the frequent interface between patients and LLMs amplifies vulnerabilities regarding data misappropriation (20, 27). A consensus across studies highlights latent safety hazards (12, 18, 26), with specific evidence (12) indicating that a lack of source transparency and information obsolescence can propagate clinical misinformation. Moreover, the issue of equity is exacerbated by heterogeneous model performance and suboptimal readability, which risks marginalizing underserved groups with restricted digital resources. accountability remains ill-defined, as the boundaries of responsibility among developers, healthcare institutions, and providers remain blurred when models generate harmful information (18, 27). These intertwined ethical dilemmas represent a formidable bottleneck for the scalable deployment of LLMs in diabetic health education.

### 4.3 Feasible suggestions for using LLMs to generate information in the field of diabetes health education

Firstly, establishing standardized evaluative metrics is paramount to ensuring the safe integration of LLMs into diabetes education. Future research must prioritize the development of a multidimensional, standardized evaluation framework coupled with objective performance metrics to facilitate robust comparisons. The TRIPOD-LLM reporting guideline (43) serves as a critical reference for establishing such standards. Furthermore, it is essential to involve real-world patients in the evaluation process. Qualitative methodologies should be employed to explore the facilitators and barriers to patient engagement with LLM-generated content, thereby enhancing model applicability.

Secondly, technological improvements to LLMs are also crucial. Researchers should employ more advanced training methods to enhance the model's generalization ability, while using more refined evaluation metrics to detect and reduce hallucinations. For example, Zhan et al. (44) improved the model's accuracy by using RAG and context-in-context learning techniques. Future research should suggest establishing a mechanism for continuous updates and alignment with external knowledge, timely integration of the latest evidence-based evidence, reducing the risk of information obsolescence, and lowering the risk of hallucinations (45). Strengthening cross-modal information processing capabilities, such as using deep fusion with knowledge graphs, can narrow the performance gap between image and text tasks (46). Finally, improving model transparency and interpretability is a key direction, including enhancing the traceability of the generation basis and information sources, thereby improving its credibility in clinical scenarios.

Ethical concerns represent the most prominent risks in integrating LLMs into the healthcare sector. Future research should align with frameworks such as the Health Insurance Portability and Accountability Act (HIPAA) (47), utilizing de-identification or anonymization of patient data and regulating the volume of identifiable information during model fine-tuning to safeguard data security. Simultaneously, the regulatory paradigm must transition from ambiguous liability definitions toward structured governance. This necessitates the acceleration of expert consensus and clinical practice guidelines to delineate the boundaries of responsibility among developers, medical institutions, and healthcare professionals, thereby integrating data governance and ethical oversight throughout the entire lifecycle of model development and implementation. Building upon this secure foundation, future research should endeavor to bridge the digital literacy divide among diverse patient populations. By leveraging fine-tuning technologies (48). Furthermore, while current outputs remain predominantly text-based, the poten (49), ultimately significantly bolstering patient acceptability and self-management self-efficacy (50).

### 4.4 Limitation

First, the inherent limitations of included studies must be acknowledged. Most current studies rely on expert-led assessments and lack rigorous clinical trials, resulting in a lack of longitudinal data to support the clinical effectiveness and real-world impact of LLMs in diabetes management. Furthermore, many studies are single-center with small sample sizes, potentially affecting the reliability and generalizability of the results. Additionally, the outcome measures in included studies rely on subjective evaluations, which may introduce potential inter-rater reliability discrepancies. This is because different raters may have significant differences in their understanding of the content generated by LLMs. Significant methodological heterogeneity exists between different study designs, limiting quantitative comparative analysis.

This study only included English literature, which may have overlooked relevant research in other languages. Most of the included studies originated from China and Turkey, with fewer reports from Africa, South Asia, and other regions. This uneven distribution of research reveals a significant lack of empirical research on LLMs in resource-scarce regions, suggesting that the cultural fit and contextual adaptability of large language models globally may be limited.

## 5 Conclusion

This scoping review elucidates the current landscape and inherent challenges of utilizing LLMs for health education in the diabetes domain. While LLMs exhibit commendable proficiency in terms of informational accuracy and comprehensiveness, they confront critical bottlenecks regarding readability and actionability. Moreover, ethical and safety concerns remain paramount. Future endeavors must prioritize patient-centered design, bolster clinical utility, and optimize technical architectures. Furthermore, the establishment of standardized outcome measures and robust ethical frameworks is essential to transition LLMs into effective, evidence-based auxiliary tools for clinical education.
