# Supplementary material for: Evaluation of large language model–generated information in diabetes health patient education: a scoping review
Source: Front Public Health. 2026 Jun 23;14:1850486. doi: 10.3389/fpubh.2026.1850486 (PMC13337864; doi:10.3389/fpubh.2026.1850486)
Supplement: Supplementary file 1 [file Supplementary_file_1.docx]

**Appendix A. PRISMA-ScR Checklist.**

| **PRISMA-ScR Item** | **Description / How addressed in this review** |
| --- | --- |
| Abstract | The title explicitly identifies this study as a scoping review of LLM applications in diabetes health education. |
| Abstract | Structured abstract includes background, objectives, eligibility criteria, sources of evidence, charting methods, main results, and conclusions. |
| Rationale | The Introduction explains the rationale for reviewing LLM applications in diabetes education, including current gaps in patient education and AI application. |
| Objectives | Objectives are clearly stated: to evaluate LLM-generated content, performance, application scenarios, and ethical challenges in diabetes health education. |
| Eligibility criteria | Inclusion and exclusion criteria are defined: studies involving LLM-generated content for diabetes patient education published in the last 4 years; excluding non-LLM studies, reviews, or studies without relevant outcome data. |
| Information sources | Relevant English literature was searched in PubMed, Embase, Web of Science, APA PsycNet, and The Cochrane Library databases using keywords such as large language model, ChatGPT, transformer model, generative AI, and diabetes. The search period was from the database inception to March 26, 2026. |
| Search strategy | Full search strategy provided in Methods section, including keywords and Boolean operators. |
| Selection of sources of evidence | Screening was conducted in two stages: title/abstract screening and full-text assessment; conflicts resolved by consensus; PRISMA-ScR flow diagram included (Figure 1). |
| Data charting process | Data extraction conducted independently by two reviewers using a standardized template, including study characteristics, LLM type, content type, application scenario, performance, and ethical considerations. |
| Data items | Data items charted: author, year, country, study design, patient population, LLM type, content type (education focus), performance metrics (accuracy, completeness, readability), and reported ethical issues. |
| Critical appraisal of individual sources | Not performed, consistent with scoping review methodology. |
| Synthesis of results | Results synthesized qualitatively; LLM performance summarized by content type and outcome measures; ethical challenges categorized. |
| Results – selection of sources of evidence | Out of 910 identified records, 269 duplicates removed, 641 screened by title/abstract, 62 full-text assessed, 41 excluded; 21 studies included; PRISMA-ScR flow diagram provided. |
| Results – characteristics of sources of evidence | Characteristics summarized in Table 1: study design, LLM type, patient population, content type, performance outcomes, and reported ethical issues. |
| Results – critical appraisal within sources | Not applicable. |
| Results – synthesis of results | Main findings summarized: LLMs perform well in accuracy and completeness but have limitations in readability, usability, and individualization; ethical risks include privacy, safety, and unclear responsibility. |
| Discussion – summary of evidence | Overview of main findings, technical and ethical implications, and potential application in clinical and patient education settings discussed. |
| Discussion – limitations | Limitations of included studies (simulation-based, small sample sizes, language/cultural limitations) and of the review itself are acknowledged. |
| Discussion – conclusions | Conclusions emphasize LLMs’ potential as an adjunctive tool, highlight technical and ethical challenges, and provide recommendations for future research, technical optimization, and structured ethical oversight. |
| Funding | Funding sources and conflicts of interest are declared in the manuscript. |
| Availability of data, code, and other materials | Data extraction tables and PRISMA-ScR diagram included in appendices; code not applicable. |

**Appendix B. Search Strategy.**

| **Database** | **Search Query and Details** | **Number** |
| --- | --- | --- |
| Pubmed | (("large language model"[All Fields] OR "LLM"[All Fields] OR "ChatGPT"[All Fields] OR "GPT-4"[All Fields] OR "GPT"[All Fields] OR "transformer model"[All Fields] OR "generative AI"[All Fields] OR "natural language generation"[All Fields])) AND ("Diabetes Mellitus"[Mesh]) | 227 |
| Web of science | TP=("large language model" OR "ChatGPT" OR "GPT-4" OR "GPT" OR "transformer model" OR "generative AI" OR "natural language generation") AND TP=(“diabetes” OR “Diabetes Mellitus”) | 373 |
| EMBASE | ('large language model':ab,ti OR 'chatgpt':ab,ti OR 'gpt 4':ab,ti OR 'gpt':ab,ti OR 'transformer model':ab,ti OR 'generative artificial intelligence':ab,ti OR 'natural language generation':ab,ti OR 'llm':ab,ti) AND 'diabetes mellitus':ab,ti | 207 |
| APA PsycNet | ("large language model" OR "ChatGPT" OR "GPT-4" OR "GPT" OR "transformer model" OR "generative AI" OR "natural language generation") AND (“diabetes” OR “Diabetes Mellitus”) | 16 |
| Cochrane Library | "large language model" OR "ChatGPT" OR "GPT-4" OR "GPT" OR "transformer model" OR "generative AI" OR "natural language generation" in Title Abstract Keyword AND "diabetes” OR “Diabetes Mellitus" in Title Abstract Keyword | 87 |
| Total |  | 910 |

**Appendix C. Ethical Challenges.**

| **Author, year** | **Ethical Challenges** |
| --- | --- |
| Subramanian et al. (26)  2024 | Potential bias |
| Chung et al. (25) 2024 | Incomplete information, unclear boundaries of responsibility |
| Tekin et al. (12) 2025 | Information obsolescence, fairness, and source transparency |
| Cheng et al. (18) 2025 | Information accuracy, security, and attribution of responsibility |
| Skjervold et al. (27) 2025 | Error message generation, data privacy and security, and liability attribution |
| Kelly et al. (28) 2025 | Error message risk, liability assignment |
| Wang et al. (19) 2024 | Interpretability, transparency, privacy, and security |
| Zha et al. (20) 2026 | Data privacy, information accuracy |
| Wang et al. (9) 2026 | Transparency, fairness |
| Hernandez et al. (22) 2023 | The information source is unreliable and lacks authoritative citation. |
| Yigit Yalcın et al. (8) 2025 | Data privacy, fairness, and patient safety risks |
| Ongen et al. (13) 2025 | Privacy risks, information accuracy |
| Sun et al. (21) 2023 | Model uncertainty, lack of transparency |
| Lindstrø et al. (6) 2024 | Data privacy, liability attribution |
| Aypar Akbağ(14) 2025 | Information source reliability |
| Bayram et al. (15) 2025 | Information accuracy, privacy risks, hallucinations, and attribution of responsibility |
| Johansen et al. (24) 2026 | Patient safety risks, model randomness |
| Şenoymak et al. (17) 2025 | Data privacy |
| Rohrich et al. (23) 2025 | Error messages, fairness |
| Goncalves et al.(29) 2026 | Information accuracy |
| Özkaya s et al.(16) 2026 | Error messages, transparency |

**Appendix D：Outcome assessment of included studies**

| **Outcome evaluated** | **Author, year** | **Evaluation modality** | **Outcomes** |
| --- | --- | --- | --- |
| Appropriateness | Kelly et al. (28) 2025 | Binary Metrics | 94% were considered perfectly appropriate, and another 6% were considered partially appropriate. |
|  | Hernandez et al. (22) 2023 | Binary Metrics | 98.5% were classified as appropriate, 1.4% were considered inappropriate, and the remaining concerns contained errors but still met the minimum standards. |
|  | Subramanian et al. (26)  2024 | Likert scales | Average score: 4.38 ± 0.55 |
| Completeness | Subramanian et al. (26)  2024 | Likert scales | Average score: 4.38 ± 0.55  This equates to an overall consensus of 87.6% on the completeness of the answers provided by artificial intelligence. |
|  | Cheng et al. (18) 2025 | Predesigned 3-point Likert scale (1 = Not comprehensive, 3 = Very comprehensive) | Expert assessment:  GPT-4o and ChatGLM4 guideline-based health education materials differ significantly from those used by physicians.  Patient assessment:  ERNIE Bot 4.0 showed no difference in performance compared to doctors. |
|  | Wang et al. (19) 2024 | Predesigned 5-point Likert scale (1 = Not comprehensive, 5 = Very comprehensive) | ChatGPT-4：4.14 ± 0.72,  RAG- ChatGPT-4: 4.69 ± 0.39,  Claude 2：3.79 ± 0.78  RAG- Claude 2：4.20 ± 0.60,  Google Bard：3.73 ± 0.80,  RAG- Google Bard: 4.10 ± 0.62 |
|  | Şenoymak et al. (17) 2025 | Predesigned 6-point Likert scale (1 = Not comprehensive, 6 = Very comprehensive) | 4.50 [4.00-5.80] |
|  | Rohrich et al. (23) 2025 | Predesigned 10-point Likert scale (1 = Not comprehensive, 10 = Very comprehensive) | 8.00 ± 0.70 |
| Comprehensibility and Actionability | Zha et al.(20)2026 | The Patient Education Materials Assessment Tool for Print Materials (PEMAT-P) | Comprehensibility：  DeepSeek R1：0.85 [0.80, 0.86],  Doubao 1.5：0.77 [0.73, 0.79],  ChatGPT-4o：0.80 [0.74, 0.85],  Actionability：  DeepSeek R1：0.80 [0.67, 1.00],  Doubao 1.5：0.80 [0.60, 1.00],  ChatGPT-4o：0.82 [0.67, 1.00] |
|  | Aypar Akbağ (14) 2025 | The Patient Education Materials Assessment Tool for Print Materials (PEMAT-P) | Comprehensibility:  ChatGPT and Gemini average: 91.36% (86.66%-93.75%),  Actionability:  ChatGPT and Gemini average: 89.67% (80%-100%) |
| Safety | Chung et al. (25) 2024 | Predesigned 4-point Likert scale (1 = Very dangerous, 4 = Very safe) | 4 |
|  | Cheng et al. (18) 2025 | Predesigned 3-point Likert scale (1 = Very dangerous, 3 = Very safe) | There was no significant difference in the safety of health education materials generated by all LLMs compared to those derived from doctors. |
|  | Zha et al. (20) 2026 | an artificial intelligence (AI) natural language evaluation tool | DeepSeek R1>ChatGPT-4o>Doubao 1.5（*P* < 0.01） |
|  | Rohrich et al. (23) 2025 | Predesigned 10-point Likert scale (1 = No dangerous, 10 = Very dangerous) | 2.20 ± 0.60 |
| Utility | Chung et al. (25) 2024 | Predesigned 4-point Likert scale (1 = Not useful, 4 = Fully useful) | 4 |
|  | Tekin et al. (12) 2025 | Predesigned 5-point Likert scale (1 = Not useful, 5 = Fully useful) | 3.00 [2.00, 3.00] |
|  | Skjervold et al. (27) 2025 | Predesigned 5-point Likert scale (1 = Not useful, 5 = Fully useful) | ChatGPT-4o's responses scored 7.2% higher than medical professionals on average |
|  | Zha et al. (20) 2026 | an artificial intelligence (AI) natural language evaluation tool | There was no statistically significant difference between DeepSeek R1, ChatGPT-4o, and Doubao 1.5（*P* > 0.01） |
| Accuracy | Wang et al. (19) 2024 | Binary Metrics | ChatGPTt-4：8.72 ± 0.70,  RAG- ChatGPTt-4:8.91 ± 0.37,  Claude 2：8.09 ± 1.23,  RAG- Claude 2：8.65 ± 0.65,  Google Bard：8.37 ± 1.36,  RAG- Google Bard :8.86 ± 0.47 |
|  | Zha et al. (20) 2026 | an artificial intelligence (AI) natural language evaluation tool | There was no statistically significant difference between DeepSeek R1, ChatGPT-4o, and Doubao 1.5（*P* > 0.01） |
|  | Sun et al. (21) 2023 | Binary Metrics | ChatGPT had an accuracy rate of 60.5% and GPT 4.0 had an accuracy rate of 74.5% |
|  | Lindstrø et al. (6) 2024 | Predesigned 5-point Likert scale (1 =Inaccurate, 5 = Highly accurate) | ChatGPT：5.00 [4.00;5.00],  DanskGPT：4.00 [3.00;4.00],  clinician’s answers：4.00 [3.00;4.00] |
|  | Bayram et al. (15) 2025 | Binary Metrics | ChatGPT-4.1 most closely matches energy requirements, achieving approximately 70.9% of the guidance target, but it overestimates fat intake.  Grok-3 energy accuracy was 83.1%, but micronutrient assessment was poor.  DeepSeek-in has high accuracy in protein analysis, but its overall energy matching accuracy is low (-63%). |
|  | Johansen et al. (24) 2026 | Binary Metrics | ChatGPT-4o achieved a PoA of 93.3% for fruits and vegetables estimates, increasing to 95% with a size reference, while composite meal estimates yielded a PoA of 46.7%, reducing to 43.3% with a size reference, based on a ±10 g carbohydrates limit ChatGPT-4o |
|  | Şenoymak et al. (17) 2025 | Predesigned 3-point Likert scale (1 = Inaccurate; 3 = Accurate). | 3.0 |
|  | Rohrich et al. (23) 2025 | Predesigned 10-point Likert scale (1 = No correct information, 10 = Only correct information) | 8.70 ± 0.30 |
|  | Goncalves et al.,(29) 2026 | Mean Absolute Error | Expert Dietitian：13 ± 10 g,  ChatGPT-4o：20 ± 18 g,  Gemini 2.5 Flash：28 ± 26g,  Claude Sonnet 4：23 ± 21g, |
|  | Özkaya s et al. (16) 2026 | Mean Absolute Error | Difference from actual carbohydrate value  ChatGPT: -5.88g (*P*=0.322),  DeepSeek: 10.58g (*P*=0.070),  Gemini: 10.99g (*P*=0.051),  Nutritionist: 4.08g (*P*=0.425) |
|  | Cheng et al. (18) 2025 | Predesigned 6-point Likert scale (1 = Very inaccurate, 6 = Very accurate) | The highest accuracy was generated by ERNIE Bot 4.0. |
|  | Kelly et al. (28) 2025 | Binary Metrics | Regarding responses that matched the literature, 73% of the answers came from the references, while 27% did not.  In the responses citing references, 94% were accurate, and 6% were partially accurate. |
|  | Chung et al. (25) 2024 | Predesigned 4-point Likert scale (1 =Completely Invalid, 4 = Highly Valid) | 28.6% rated 3 points, 71.4% rated 4 |
|  | Skjervold et al. (27) 2025 | Predesigned 5-point Likert scale (1 = Completely Invalid, 5 =Highly Valid) | ChatGPT-4o responses received an average score 7.9% higher than those from medical professionals. |
|  | Subramanian et al. (26)  2024 | Predesigned 5-point Likert scale (1 = Not comprehensive, 5 = Very comprehensive) | Average score: 4.84± 0.33  This corresponds to an overall agreement rate of 96.8%compared to the expert evaluations. |
| Quality | Tekin et al. (12) 2025 | The Global Quality Scale (GQS),  DISCERN scale | GQS  4.00 [3.00, 5.00]  DISCERN scale  28.40 ± 1.60 |
|  | Yigit Yalcın et al. (8) 2025 | The Global Quality Scale (GQS),  DISCERN scale | GQS  ChatGPT：3.86 ± 0.76,  DeepSeek：4.21 ± 0.76,  Gemini：4.41 ± 0.66,  Grok：4.27 ± 0.90  DISCERN scale  ChatGPT：25.40 ± 4.18,  DeepSeek：27.23 ± 4.55,  Gemini：28.49 ± 4.36,  Grok：29.01 ± 6.01 |
|  | Ongen et al. (13) 2025 | The Global Quality Scale (GQS),  DISCERN scale | GQS  ChatGPT 3.5：3.40 ± 1.30,  ChatGPT 4o：3.78 ± 1.09,  ChatGPT 4：3.72 ± 1.12,  Gemini: 3.40 ± 1.24,  Gemini Advanced：3.42 ± 1.27  DISCERN scale |
|  | Wang et al. (9) 2026 | The Global Quality Scale (GQS)  The Ensuring Quality Information for Patients (EQIP) instrument，  DISCERN scale | GQS:  ChatGPT: 4.00 ± 0.65,  Claude Sonnet: 3.27 ± 0.80,  DeepSeek: 3.60 ± 0.74,  Gemini: 3.27 ± 0.80.  EQIP:  ChatGPT: 71.67 ± 6.17,  Claude Sonnet: 59.00 ± 6.87,  DeepSeek: 66.00 ± 5.07,  Gemini: 61.67 ± 5.88  DISCERN scale  ChatGPT: 42.53 ± 7.20,  Claude Sonnet: 30.67 ± 7.37,  DeepSeek: 34.80 ± 6.10,  Gemini: 32.67 ± 8.30 |
| Readability | Tekin et al. (12) 2025 | Flesch Reading Ease (FRES),  Flesch-Kincaid Grade Level (FKGL),  The Gunning Fog Index (GFI) | FRES :37.80 ± 13.50,  FKGL： 10.50 ± 2.26,  GFI： 11.10 ± 3.03 |
|  | Wang et al. (19) 2024 | Predesigned 5-point Likert scale (1 = Unreadable, 5 = Highly readable) | ChatGPT：4.32 ± 0.61,  RAG- ChatGPT:4.64 ± 0.51,  Claude 2：4.01 ± 0.73,  RAG- Claude 2：4.07 ± 0.74,  Google Bard：3.96 ± 0.86，  RAG- Google Bard: 4.10 ± 0.62 |
|  | Wang et al. (9) 2026 | The Automated Readability Index (ARI), Coleman–Liau Index (CL),  Flesch–Kincaid Grade Level (FKGL),  Flesch Reading Ease score (FRES),  Gunning Fog Index (GFI),  Simple Measure of Gobbledygook (SMOG index) | ARI:  ChatGPT-5: 8.06 ± 2.42,  Claude Sonnet 4.5: 14.11 ± 3.85,  DeepSeek -V3.2: 11.43 ± 2.41,  Gemini 2.5 Pro: 14.32 ± 2.02.  CL:  ChatGPT-5: 9.62 ± 2.37,  Claude Sonnet: 15.70 ± 2.76,  DeepSeek -V3.2: 12.75 ± 1.80,  Gemini 2.5 Pro: 13.42 ± 1.53.  FKGL:  ChatGPT-5: 7.43 ± 2.42,  Claude Sonnet 4.5: 13.11 ± 3.54,  DeepSeek -V3.2: 9.64 ± 2.42,  Gemini 2.5 Pro: 13.04 ± 1.96.  FRES:  ChatGPT-5: 62.47 ± 13.51,  Claude Sonnet 4.5: 29.33 ± 17.36,  DeepSeek -V3.2: 51.40 ± 11.65,  Gemini 2.5 Pro: 38.67 ± 10.39.  GFI  ChatGPT-5: 9.41 ± 2.05,  Claude Sonnet 4.5: 14.91 ± 2.86,  DeepSeek -V3.2: 11.23 ± 2.01,  Gemini 2.5 Pro: 13.55 ± 1.86.  SMOG:  ChatGPT-5: 7.78 ± 1.56,  Claude Sonnet 4.5: 11.75 ± 2.60,  DeepSeek -V3.2: 10.23 ± 1.73,  Gemini 2.5 Pro: 11.97 ± 1.55. |
|  | Yigit Yalcın et al. (8) 2025 | Flesch Reading Ease (FRES),  Flesch–Kincaid Grade Level (FKGL),  The Gunning Fog Index (GFI),  Coleman–Liau Index (CLI),  Simple Measure of Gobbledygook (SMOG index) | FRES  ChatGPT：39.42 ± 11.96,  DeepSeek：49.53 ± 7.68,  Gemini：42.64 ± 9.32,  Grok：27.66 ± 11.52.  FKGL  ChatGPT：15.86 ± 3.83,  DeepSeek：11.04 ± 1.65,  Gemini：12.03 ± 1.89,  Grok：17.06 ± 3.52.  SMOG index  ChatGPT：15.37 ± 2.32,  DeepSeek：12.76 ± 1.42,  Gemini：14.01 ± 1.62,  Grok：17.21 ± 2.29.  GFI  ChatGPT：18.94 ± 3.95,  DeepSeek：13.88 ± 1.96,  Gemini：15.33 ± 2.24,  Grok：20.44 ± 3.65.  CLI  ChatGPT：10.90 ± 1.78,  DeepSeek：10.77 ± 1.95,  Gemini：12.76 ± 1.47,  Grok：13.71 ± 1.32.  TTR  ChatGPT：0.58 ± 0.04,  DeepSeek：0.60 ± 0.04,  Gemini：0.45 ± 0.03,  Grok：0.41 ± 0.08 |
|  | Aypar Akbağ (14) 2025 | The Ateşman Readability Formula  The Gunning Fog Index (GFI) | The Ateşman Readability Formula：  ChatGPT：68.8  Gemini：69.4  GFI：  ChatGPT：18.57  Gemini：17.54 |
|  | Rohrich et al. (23) 2025 | Flesch Reading Ease (FRES),  Flesch-Kincaid Score,  The Gunning Fog Index (GFI),  Coleman–Liau Index (CLI),  Simple Measure of Gobbledygook (SMOG index)  The Automated Readability Index (ARI),  Linsear Write Formula, New Dale-Chall Score | FRES：3.80 ± 2.60  GFI：14.70 ± 2.60  Flesch-Kincaid Score：11.90 ± 2.20  CLI：14.00 ± 1.90  SMOG index：14.10 ± 1.80  ARI：12.50 ± 2.50  Linsear Write Formula：12.80 ± 2.80  New Dale-Chall Score ：6.80 ± 0.90  Grade Level：11.90 ± 1.80 |
| Empathy | Skjervold et al. (27) 2025 | Predesigned 5-point Likert scale (1 =Dismissive, 5 =Highly Empathetic) | Chat-GPT-4o responses received an average score 10.1% higher than those from medical professionals. |
| Overall patient assessment | Cheng et al. (18) 2025 | blinded manner | ERNIE Bot 4.0 performed on par with physicians in terms of patient comprehensibility, completeness, and safety. 60% (30/50) of patients felt that ERNIE Bot 4.0 performed as well or better than the physician |
| Personalization | Zha et al. (20) 2026 | an artificial intelligence (AI) natural language evaluation tool | There was no statistically significant difference between DeepSeek R1, ChatGPT-4o, and Doubao 1.5（*P* > 0.01） |
